# Comment on Scheepers et al. Comparative Performance Testing of Respirator versus Surgical Mask Using a Water Droplet Spray Model. *Int. J. Environ. Res. Public Health* 2021, *18,* 1599

**DOI:** 10.3390/ijerph19116353

**Published:** 2022-05-24

**Authors:** Andrew Viner, Stewart Ayrey

**Affiliations:** 1 3M Personal Safety Division, St. Paul, MN 55144, USA; andrewviner@mmm.com; 2 3M Personal Safety Division, 3M United Kingdom PLC, Bracknell RG12 8HT, UK

In the article “Comparative Performance Testing of Respirator versus Surgical Mask Using a Water Droplet Spray Model” by Scheepers et al. [1], the authors propose a new method for evaluating the performance of an FFP2 respirator and surgical mask. Upon careful review of the article, we find that shortcomings in their approach leave us concerned that readers may draw incorrect conclusions about the protection that a healthcare worker may obtain by wearing a surgical mask rather than a respirator.

Our first concern is with the use of the term Total Inward Leakage to describe the results of their measurements. Total Inward Leakage is a term of art that has been used in the field of respiratory protection for decades to refer to the leakage of a respirator measured in a specific way. The authors cite European Standard EN 149:2001 [2] for the definition of Total Inward Leakage (TIL) (see Clause 7.9.1 of that document); however, they overlook the last sentence in that Clause: “Testing shall be done in accordance with 8.5”. Those familiar with EN standards will recognize that the word *shall* indicates a mandatory requirement. Clause 8.5 of EN 149:2001 provides detailed instructions on the proper measurement of TIL, which include the use of an aerosol of sodium chloride particles, a flame photometer for the measurement of the NaCl concentration, and human subjects walking on a treadmill in a chamber with a uniformly distributed, stable concentration of test aerosol. Insofar as the test method described in Scheepers et al. includes none of those elements, it is inappropriate for them to call their results “Total Inward Leakage”. In fact, there is no way to compare the results reported by the authors with properly measured TIL values because their results consist only of the mass of fluorescein captured on the mouth filter (expressed with units of nanograms). Clause 8.5.2.3 of EN 149:2001 provides a formula defining leakage as the ratio of the concentration of challenge agent measured inside and outside of a respirator, corrected for the subject’s breathing pattern and expressed as a percentage. A close reading of the article is required in order to understand that the values they report bear no relationship to TIL as measured according to EN 149. To that extent, the authors have done a disservice to the respiratory protection community (no doubt unintentionally), by adopting the term TIL and thereby introducing confusion into the literature.

Our second objection arises from the use of a dummy head for evaluating the leakage of respirators and surgical masks. While the authors acknowledge the importance of a respirator design with a good fit to protect against smaller aerosols, they overlook or are unaware of several limitations to the use of a dummy head for evaluating how well a mask or respirator will fit on a human. The authors describe the dummy head as “anatomically correct”; however, the photo in their Figure 2b shows that the dummy head does not have ears. Proper placement of the head straps of a respirator or mask may rely on the placement of straps above the ears. In their Figure 2a, it would appear that the placement of the upper strap on the dummy head is too low because it cannot rest above an ear. Further, the authors state, “The RPEs to be tested were placed on the dummy head according to the instructions provided by the supplier.” However, the instructions provided by respirator manufacturers are intended for humans to don respirators on themselves. The human wearer has the advantage of tactile feedback to aid in properly positioning a respirator on their face, forming the noseclip with the appropriate technique and amount of pressure, and, just as importantly, conducting a user seal check to verify that there are no leaks. A test operator donning a respirator onto a dummy head lacks the benefit of those feedback mechanisms. To understand the importance of these elements of respirator donning, one should try this experiment: sit or stand quietly while another person tries to don a respirator on them. It is quite unlikely that anyone would be satisfied with the fit of a respirator that was donned on them by a colleague, no matter how well-meaning they were.

Perhaps the greatest limitation in using dummy heads to assess how well a respirator fits on a human face is the rigid nature of most dummy heads. When a human dons a respirator, the pressure applied by the headbands causes the face to deform in the cheek and chin areas and, to a lesser extent, around the nose and under the eyes. The amount of deformation depends on the size of a person’s head, the length and elasticity of the headbands on the respirator, and, perhaps most importantly, the amount of tissue making up a person’s face. That deformation helps make a seal of the perimeter of the respirator against the face. By comparison, the face on a rigid dummy head cannot deform in response to the pressure applied by the headbands, so significant leaks between the respirator and dummy head are almost guaranteed.

Researchers at the National Institute for Occupational Safety and Health (NIOSH), part of the Centers for Disease Control, attempted to address these limitations by developing an advanced headform that included a deformable face surface [3]. They conducted an extensive study to compare the fit factors of respirators worn first by humans and subsequently donned on the advanced headform [4]. The fit factor is a metric that is similar but not directly related to TIL. They took several steps to try to achieve the same fit on the headform as the one that was measured on the human, including measuring each test subject’s breathing pattern and then replicating that pattern with a breathing machine attached to the headform, and performing a simulated user seal check prior to conducting the fit tests. For all eight models of N95 respirators that they tested, the average fit factor measured on humans was greater than (i.e., better than) that measured on the headform. For four of the eight respirator models, the difference was statistically significant. In spite of their best efforts, fits measured on the headform frequently underpredicted the fit attained on humans, in many cases by orders of magnitude. There is no reason to believe that the magnitude of errors associated with the measurement of TIL on a rigid dummy head would be any different. Given the difficulties that NIOSH researchers experienced in attempting to match the fit measured on humans with the fit measured on a lifelike headform, we believe that the results reported by Scheepers and co-workers do not accurately reflect how well a product would fit on a human face. Therefore, we caution against concluding that a surgical mask can provide protection equivalent to or better than a respirator.

Third, we feel compelled to correct an apparent misconception about the filtration of bioaerosols. The authors state that the need for their new test method arose from concerns that the test methods described in EN 149:2001 do not take into account the “different properties” of bioaerosols released in clinical settings. There is a large body of evidence in the literature showing that the nature of the particle is irrelevant to the performance of a filter. For example, filter penetration measurements of various NIOSH-approved respirators with both inert particles and bioaerosols found that for particles of the same size, the filtration performance was not affected by the type of particle [5]. Bioaerosols behaved the same as inert particles. In 2014, NIOSH published a blog post titled “Do We Need to Challenge Respirator Filters with Biological Aerosols?”, which summarized literature comparing filter performance measured with inert particles and bioaerosols [6]. Their conclusion is that particle capture depends on the size, shape, and density of a particle; the nature of the particles, whether they are viable bioaerosol or inert particles, makes no difference. NIOSH subsequently published a video [7] that made the case that “a particle is a particle”.

Furthermore, the inclination to test with bioaerosols can lead to inappropriate conclusions about the performance of a filter. For example, the Bacterial Filtration Efficiency (BFE) test (ASTM F2101 [8] and EN14683—Annex B [9]) that is specified for testing surgical masks uses *Staphylococcus aureus* particles, which are nominally 3 μm in diameter. The NaCl particles specified for testing NIOSH N95 respirators and filtering facepiece respirators in EN 149:2001 are much smaller than that and much closer to the most penetrating particle size of the filters. Rengasamy et al. [10] published an in-depth comparison of the performance of N95 respirators, surgical masks, and surgical N95 respirators based on BFE, N95, and other tests. All the products tested were more than 97% efficient at removing the bioaerosol, whereas when tested with the NIOSH NaCl filtration test, only the N95 products had efficiencies above 95%. The surgical masks yielded efficiencies as low as 54%. In describing these results, the authors commented, “Products with poor or mediocre filtration performance such as the SM [surgical mask] models in this study can be consistently identified using only a more challenging method such as the NIOSH NaCl method used for NIOSH certification of particulate respirators”.

In summary, the authors used the term Total Inward Leakage inappropriately, and their results cannot be compared to TIL values measured according to EN 149. In addition, their measurements only indicate how respirators and surgical masks fit on rigid dummy heads. We urge caution for anyone attempting to draw conclusions about how respirators and surgical masks fit on humans, and therefore provide protection to the wearer, based on those results.
